# Gluten-Induced Pancytopenia: A Pediatric Case Report and Literature Review

**DOI:** 10.7759/cureus.89681

**Published:** 2025-08-09

**Authors:** Khalil Elouadghiri Fouad, Ayad Ghanam, Amal Hamami, Anass Haloui, Abdeladim Babakhouya, Maria Rkain

**Affiliations:** 1 Department of Pediatrics, Faculty of Medicine and Pharmacy, Mohamed First University, Oujda, MAR; 2 Department of Pediatrics, Mohammed VI University Hospital Center, Oujda, MAR; 3 Department of Pathology, Mohammed VI University Hospital Center, Oujda, MAR; 4 Department of Pathology, Faculty of Medicine and Pharmacy, Mohamed First University, Oujda, MAR

**Keywords:** celiac disease, gluten-free diet, malabsorption, pancytopenia, vitamin b12

## Abstract

We report the case of a nine-year-old boy who presented with severe pancytopenia and respiratory distress. His medical history was notable for pica, chronic epigastric pain, pallor, and intermittent vomiting. Initial laboratory investigations revealed profound anemia (Hemoglobin (Hb) 2 g/dL), neutropenia, thrombocytopenia, and significant deficiencies in vitamin B12 and vitamin D. Serologic testing was positive for anti-transglutaminase IgA antibodies, and duodenal biopsies confirmed celiac disease (CD) (Marsh stage 3a), along with *Helicobacter pylori* gastritis. Management consisted of a strict gluten-free diet, vitamin supplementation, and *H. pylori* eradication therapy. Over the following year, the patient demonstrated marked clinical improvement, with normalization of hematologic parameters, nutritional status, and growth. This case highlights the importance of considering CD in the differential diagnosis of unexplained pancytopenia in pediatric patients, even in the absence of overt gastrointestinal symptoms.

## Introduction

Celiac disease (CD) is a systemic autoimmune disorder triggered by the ingestion of gluten proteins in genetically predisposed individuals. It is characterized by reversible inflammation of the small intestinal mucosa, with villous atrophy leading to malabsorption [1]. Historically, CD was diagnosed primarily in patients presenting with overt signs of malabsorption, such as diarrhea, steatorrhea, weight loss, or failure to thrive, along with abdominal distension, muscle wasting, or multiple deficiencies of both macronutrients and micronutrients, the so-called classic form. It is now well recognized that many patients, if not the majority, may present with much subtler symptoms, commonly referred to as atypical CD, and often without the classic features of malabsorption or even diarrhea [2]. CD may also present with a wide spectrum of extraintestinal manifestations, including rheumatologic, neurologic, endocrine, dermatologic, and hematologic findings. Various hematologic abnormalities, such as anemia, thrombocytopenia, thrombocytosis, thrombotic or hemorrhagic events, IgA deficiency, and splenic dysfunction, have been reported. Nutritional deficiencies and autoimmunity are thought to underlie these presentations [2]. Pancytopenia associated with CD has been reported only rarely [3]. Here, we report a new case of pancytopenia revealing underlying CD in a nine-year-old child. Our aim is to raise clinician awareness of this rare association and encourage consideration of CD in pediatric patients presenting with unexplained hematologic manifestations.

## Case presentation

A nine-year-old boy, born from a non-consanguineous marriage, with a history of pica (geophagy) since the age of four, chronic epigastric pain, pallor, and occasional vomiting since age five, presented to the emergency department with respiratory distress that had worsened over the previous two days. On clinical examination, he was drowsy, pale, tachycardic at 150 beats per minute, tachypneic at 38 breaths per minute, with peripheral oxygen saturation of 87% on room air, and afebrile. There were no signs of shock or peripheral circulatory failure. He had dry skin and digital clubbing. His weight was 21 kg (-2 SD), and his height was 129 cm (-1 SD). Pleuropulmonary and cardiovascular examinations were unremarkable, and no evidence of tumor syndrome was found. Laboratory results showed neutropenia and monocytosis, along with severe macrocytic anemia, thrombocytopenia, and a moderate reticulocytosis. The blood smear was unremarkable. Liver function tests and renal function were normal. Serum calcium was low, while potassium, sodium, and phosphate levels were within normal limits. Prothrombin time was slightly reduced. Given the clinical signs of severe anemia, the child received a transfusion of red blood cell concentrates. Pre-transfusion vitamin assays revealed a severe vitamin B12 deficiency, with normal vitamin B9 levels. Additional tests for malabsorption showed vitamin D deficiency, marked hypoproteinemia, and hypoalbuminemia. The lipid profile was significantly decreased. Anti-transglutaminase IgA antibodies were positive. The detailed laboratory results are presented in Table 1.

**Table 1:** Laboratory findings of the patient. WBC, white blood cells; MCV, mean corpuscular volume; MCHC, mean corpuscular hemoglobin concentration; ALAT, alanine aminotransferase; ASAT, aspartate aminotransferase

Parameter	Result	Reference Range
Complete blood count		
WBC	4,890/µL	4,000-10,000/µL
Neutrophils	990/µL	1,500-7,500/µL
Monocytes	740/µL	100-1,000/µL
Hemoglobin	2 g/dL	11.5-15.5 g/dl
MCV	111 fL	80-95 fL
MCHC	33%	32-36%
Platelets	73,000 /µL	150,000-400,000/µL
Reticulocytes	76,400 /µL	100,000-120,000/µL
Liver function tests		
ALAT	21 IU/L	<35 IU/L
ASAT	19 IU/L	<35 IU/L
Renal function		
Urea	0.2 g/L	0.10-0.30 g/L
Creatinine	6 mg/L	3.9-7.3 mg/L
Electrolytes		
Serum calcium	69 mg/L	88-105 mg/L
Serum potassium	4.1 mEq/L	3.4-4.7 mEq/L
Serum sodium	136 mEq/L	135-145 mEq/L
Phosphate	51 mg/L	40-65 mg/L
Coagulation		
Prothrombin activity	68%	70-100%
Vitamins		
Vitamin B12	140 pg/mL	200-900 pg/mL
Vitamin B9 (folates)	7 ng/mL	4-20 ng/mL
Vitamin D	6.8 ng/mL	>20 ng/mL
Proteins and albumin		
Total protein	56 g/L	65-80 g/L
Albumin	32 g/L	35-50 g/L
Lipid profile		
Total cholesterol	0.5 g/L	1.5-2 g/L
Triglycerides	0.85 g/L	0.5-1.5 g/L
Celiac serology		
Anti-transglutaminase IgA	97 IU/mL	<10 IU/mL

**Figure 1:**
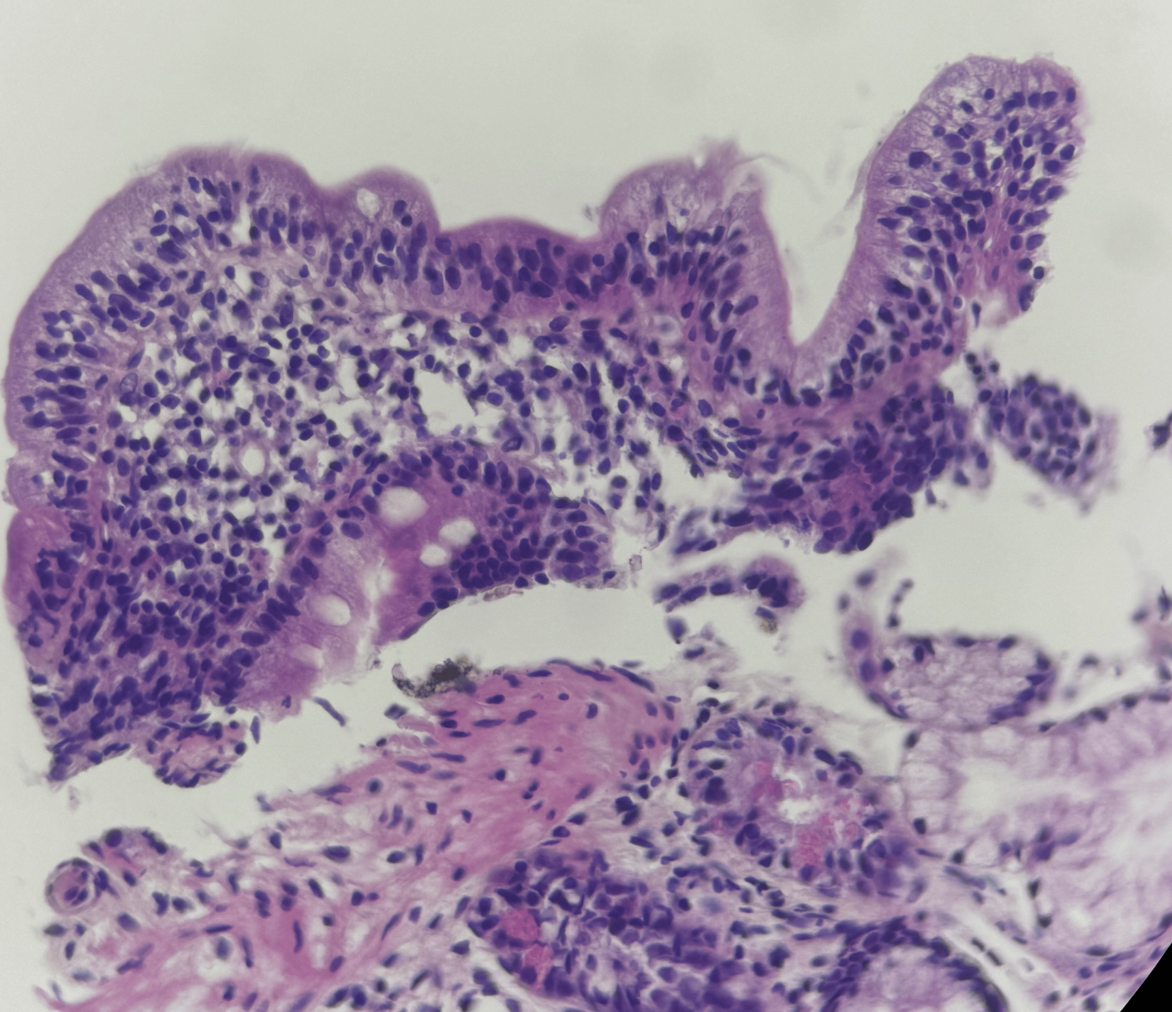
Duodenal biopsy (H&E stain, high magnification) showing villous atrophy with epithelium infiltrated by numerous intraepithelial lymphocytes (48 per 100 enterocytes), typical of CD. The lamina propria is infiltrated by a lymphoplasmacytic infiltrate. H&E, hematoxylin and eosin; CD, celiac disease

**Figure 2:**
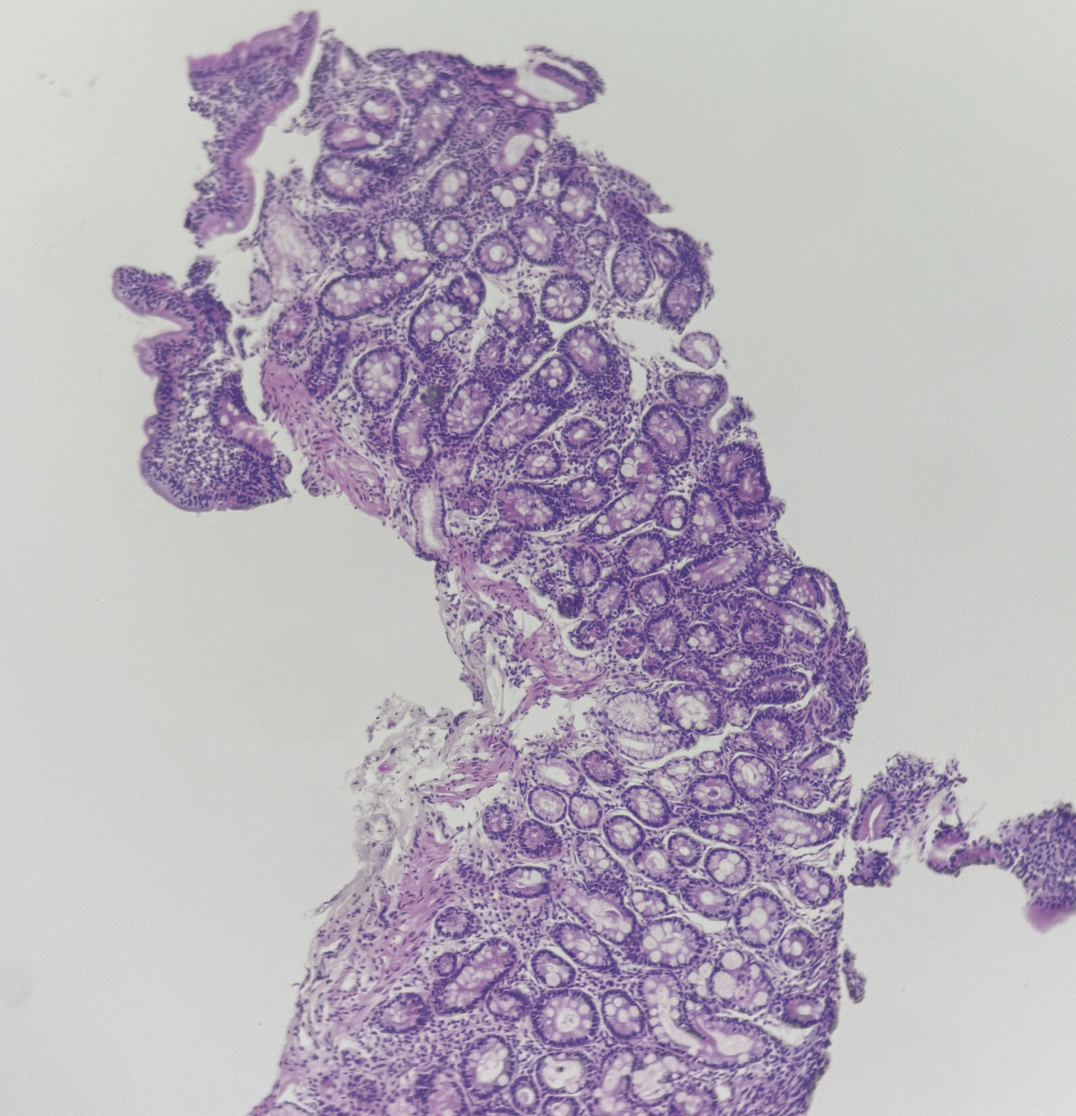
Duodenal biopsy (H&E stain, low magnification) showing marked villous atrophy associated with crypt hyperplasia. The villous-to-crypt ratio is reversed (<1), consistent with Marsh stage 3a CD. H&E, hematoxylin and eosin; CD, celiac disease

An esophagogastroduodenoscopy (EGD) with biopsies from the fundus, antrum, angulus, and duodenum was performed. Histopathological analysis confirmed the diagnosis of CD, classified as stage 3a according to the MARSH classification (Figure 1 and Figure 2), along with *Helicobacter pylori* gastritis.

The child received *H. pylori* eradication therapy along with vitamin B12 supplementation via daily intramuscular hydroxocobalamin injections (1000 µg) for one week, followed by weekly injections for one month, and then monthly. A strict gluten-free diet was introduced, along with vitamin D supplementation. Under the gluten-free diet, the child showed favorable progress, with an 8 kg weight gain and a 10 cm height increase over one year. Full normalization of the complete blood count (CBC), lipid profile, and calcium levels was achieved.

## Discussion

CD is an autoimmune disorder triggered by gluten consumption in genetically predisposed individuals, occurring in those who carry HLA class II molecules of the DQ2 or DQ8 type [4]. CD often presents with a wide range of symptoms and, in many cases, manifests atypically with only extraintestinal symptoms [5]. The diagnosis of CD is typically confirmed through a small intestine biopsy. The histopathological detection of villous atrophy in the intestinal mucosa remains the gold standard for diagnosis [2]. Serological diagnosis relies on detecting IgA antibodies against gliadin, endomysium, and tissue transglutaminase. A recent systematic review of the diagnostic accuracy of available antibody tests showed that the sensitivity of anti-endomysium (anti-EMA) and anti-tissue transglutaminase (anti-tTG) antibodies exceeds 90% [6]. Anti-tTG antibodies are considered the most practical and are widely used in diagnosing CD [2]. The coexistence of IgA deficiency in 3%-5% of patients with CD presents challenges in interpreting serological test results. It is, therefore, recommended to measure total IgA levels before ruling out the diagnosis in the presence of negative serological tests. In such cases, IgG anti-tTG antibodies may be helpful for diagnosis [2]. Due to their low sensitivity and specificity, anti-gliadin antibodies are less commonly used [7]. Various hematologic disorders can occur in CD, including anemia, thrombocytosis, thrombocytopenia, leukopenia, neutropenia, venous thromboembolism, coagulopathy, hyposplenism, IgA deficiency, and intestinal lymphoma, with anemia being the most frequent [2]. Pancytopenia, however, is rarely reported [3]. Pancytopenia in CD may arise from deficiencies in nutrients such as vitamin B12, folate, and copper, or from autoimmune processes leading to aplastic anemia with hypoplastic bone marrow [8]. Vitamin B12 and other micronutrient deficiencies in patients with CD are likely multifactorial, involving several interconnected mechanisms. A primary factor is reduced oral intake, often associated with anorexia and vomiting, which are frequent clinical manifestations of this autoimmune enteropathy. Another major contributor is intestinal malabsorption due to villous atrophy, which significantly decreases the absorptive surface area and impairs the functional capacity of the enterocyte brush border. Furthermore, increased intestinal losses of micronutrients may occur as a result of accelerated epithelial turnover induced by chronic inflammation of the small intestinal mucosa [2]. In untreated CD, folate deficiency ranges from 18% to 90%, while vitamin B12 deficiency ranges from 12% to 41% [8].

In our patient, pancytopenia was attributed to vitamin B12 deficiency. Considering the presence of other indicators of malabsorption and growth retardation, bone marrow aspiration was deemed unnecessary. When pancytopenia is caused by vitamin deficiencies, treatment is primarily based on correcting the specific nutritional deficits and implementing a strict gluten-free diet. Supplementation with folic acid and vitamin B12 is the mainstay of treatment, with the intramuscular route recommended for vitamin B12 administration [9]. Clinical and hematological improvement is generally observed within weeks of initiating therapy, provided that adherence to the gluten-free diet is strict [2]. Beyond nutritional deficiencies, pancytopenia may also result from an autoimmune mechanism. Although rare, this manifestation has been reported in several pediatric cases in which bone marrow biopsy revealed hypocellular marrow in the absence of malignant infiltration [2]. Active destruction of hematopoietic bone marrow cells mediated by T lymphocytes has been observed, with DNA damage followed by apoptosis triggered by gluten ingestion in genetically predisposed individuals [10]. These patients respond well to a gluten-free diet alone, in contrast to adults, who often require immunosuppressive therapy (such as anti-thymocyte globulin or cyclosporine) or hematopoietic stem cell transplantation [8]. The shorter duration of exposure to autoimmune processes in children compared to adults likely contributes to better outcomes, as early implementation of a gluten-free diet reduces antigenic stimulation and allows for reversal of the pathological process.

## Conclusions

Unexplained pancytopenia may represent an atypical hematologic manifestation of CD, particularly in pediatric patients. Given the potential for complete clinical and hematologic remission following the initiation of a strict gluten-free diet, CD should be systematically considered in the differential diagnosis of idiopathic pancytopenia, even in the absence of gastrointestinal symptoms. Early diagnosis enables targeted, non-immunosuppressive intervention and may prevent progression to more severe complications.
